# Vaximap: route optimisation for housebound vaccination

**DOI:** 10.1038/s41746-022-00726-2

**Published:** 2022-12-16

**Authors:** Thomas F. Kirk, Adam J. Barker, Armen Bodossian, Robert Staruch

**Affiliations:** 1 Institute of Biomedical Engineering, Department of Engineering Science, University of Oxford, Oxford, England; 2 Sir Peter Mansfield Imaging Centre, School of Medicine, University of Nottingham, Nottingham, England; 3 Squarepoint Capital LLP, London, England; 4 Visa Inc., London, England; 5 Defence Deanery, Academic Department of Military Surgery and Trauma, Birmingham, England

**Keywords:** Preventive medicine, Health care economics

## Abstract

During the United Kingdom’s Covid-19 vaccination campaign, general practitioners (GPs) have held responsibility for vaccinating housebound patients. This presented them with a large, complex and unfamiliar logistical challenge, namely determining the most time-efficient route to visit multiple patients at their home address. In response to a lack of existing solutions tailored specifically to vaccination, and in light of overwhelming demand, Vaximap (https://www.vaximap.org) was created in January 2021 to automate the process of route planning. It is free of charge for all users and has been used to-date to plan vaccinations for over 470,000 patients. This article analyses usage data to estimate the time savings (3 work years) and financial savings (£110,000) the service has yielded for GP surgeries, thus demonstrating that it helped to accelerate the UK’s Covid-19 vaccination campaign at critical moments.

## Introduction

The UK’s vaccination campaign against SARS-Cov-2 was launched in early 2021. One of the first questions to be addressed was to determine how severely limited quantities of vaccine should be allocated across the population, in order to most quickly reduce the severity of the disease. The Joint Committee on Vaccination and Immunisation (JCVI) identified nine priority groups of descending age and risk order^1^; the top four groups, including 3.7 million clinically extremely vulnerable individuals^2^, were to be vaccinated by the 15th February 2021, just six weeks into the wider campaign^3^. Within the top four groups, it is estimated that around half a million individuals were housebound (numerous freedom of information requests to NHS England, Public Health England and the JCVI have not yielded a precise figure, and correspondence with a member of the Health Informatics Group at the Royal College of General Practitioners suggests a figure of around 500,000 with a lower bound of 250,000), meaning that vaccination would need to take place at the patient’s home. Responsibility for vaccinating these patients was assigned to GPs, thus presenting primary care with a complex and unfamiliar logistical challenge of a scale wholly unprecedented in recent history.

Even before the pandemic created additional pressures, primary care in the UK was stretched and under-resourced^4,5^. GP numbers have been declining since 2015 and there exists substantial regional variations in primary care accessibility: for example, there are ~3000 patients per GP in Luton, compared to 1770 per GP in the Vale of York^6,7^. The burden imposed upon GPs by the additional responsibility to plan and deliver vaccinations to housebound patients thus fell asymmetrically across primary care. Recognising that this task would stretch already-limited resources, NHS England allocated additional funding to GPs in February 2021 specifically to support the vaccination of housebound individuals and ensure the wider campaign could proceed on schedule^3^. Crucially, however, no specific technical solution was put in place to support housebound vaccination, an issue that was raised by individual GPs on social media discussions, alongside the fact that they had not been given any training for a task that was completely new to them. In short, the funds had been allocated, but not the means. In response to these concerns from clinicians, Vaximap was created to automate the process of route planning.

**Fig. 1:**
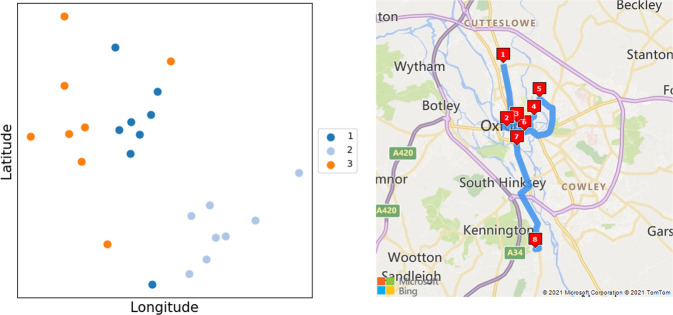
Example output produced by Vaximap. Left: 24 patients have been sorted into three clusters of size eight, the spatial distribution of which is shown here. Right: the optimal driving route for visiting the patients in cluster 1 (dark blue). [Map reproduced with permission from Microsoft under Bing Maps API Print Rights, non-profit use^27^.].

The central problem in optimising the vaccination of housebound patients is determining the fastest order in which to visit the individuals. When the route must start and end at the same location (a GP surgery, for example), this is the travelling salesman problem (TSP), or, when the start and end points are unrestricted, this is the vehicle routing problem (VRP); both problems have been extensively studied in the domains of computer science and operational research. Notwithstanding numerous solutions to these problems^8–11^, some of which have been applied in a medical context^12^, vaccination imposes an extra constraint: as there are a fixed number of doses in a vial, it is preferable to sort patients into groups of this same number. This ensures that exactly one vial (or integer multiples thereof) of vaccine will be required per group of visited patients. Separately, due to the cold-storage requirements of the vaccines themselves, visiting patients in the fastest order minimises the time that vials spend outside of the cold chain. Taken together, observing these two principles reduces vaccine wastage, especially important given the limited availability of supplies during the early stages of the campaign^13^. Vaximap operates in a two-stage manner: first clustering patients into groups of a fixed size, and then finding the optimal route within each group, which is illustrated in Fig. 1.

The creation of Vaximap was a thoroughly grass-roots effort to tackle the challenge of housebound vaccination. The initial request for help came from a GP in Wales, who subsequently tested the first prototype. After 48 hours, the production version was deployed online and thereafter was shared entirely through word of mouth within the GP community. At no point did a central authority within the NHS support or encourage the uptake of this tool. Of vital importance was a strict data-management policy: Vaximap requires a bare minimum of non-identifying patient data (postcodes) in order to function. This was crucial to satisfy GP’s justified caution in adopting a new technology. Within a month of Vaximap’s launch, 100,000 vaccine deliveries had been planned on the site and the service was adopted for use by roving military vaccination teams during operation RESCRIPT, the UK military’s support to HM Government during the pandemic.

The purpose of this work is threefold. First, to highlight the rapid deployment of a free digital technology to meet time-sensitive vaccination requirements. Secondly, to explain how the service functions and, finally, to estimate the benefits arising from its use thus far. As it would be methodologically challenging to quantify the benefits in terms of health outcomes, they are instead quantified in terms of time and cost savings. As of October 2022, we estimate that 285,000 home vaccinations have taken place as a result of using the service, representing about 50% of the target patient population, which represents time and cost savings of ~3 practitioner-years and £110,000 respectively.

## Results

### Summary metrics of use

**Fig. 2:**
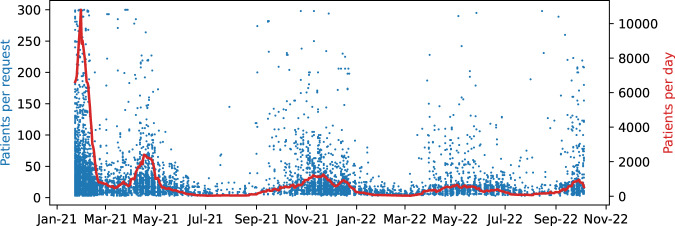
Time series of Vaximap use. Peaks assumed to correspond to the majority of first (Jan 21) and second doses (Apr 21) can be discerned, as can booster doses (Nov 21, May 22 and Oct 22). The red line is the 30-day moving average of total uploaded patients per day. A few uploads of the maximum permitted number of patients (300 patients) can be observed.

Figure 2 shows the time series of Vaximap use, both in terms of total number of daily patients, and patients per user request. Five peaks can be seen: February and April 2021 correspond to initial doses, whilst November 2021, May and October 2022 correspond to subsequent booster doses.

**Fig. 3:**
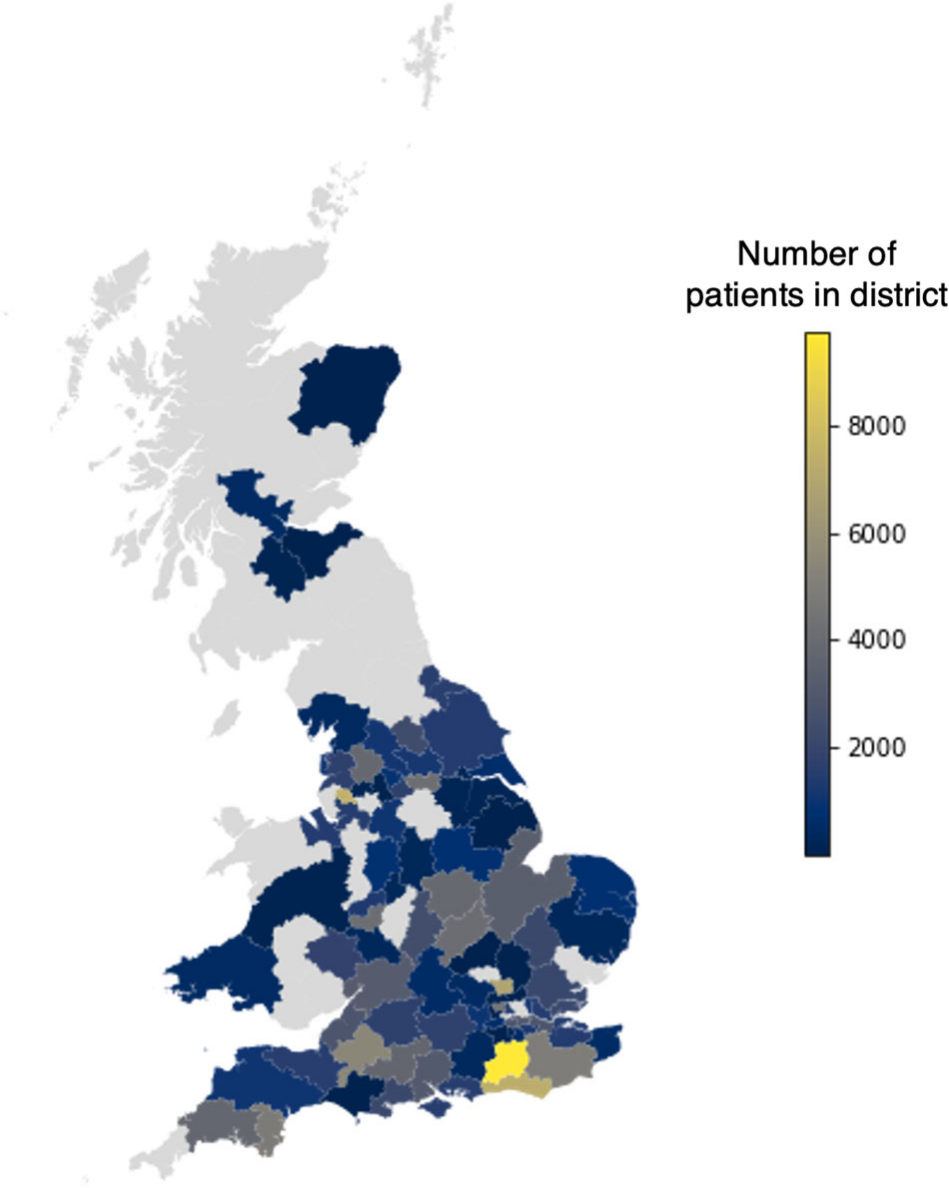
Geographic distribution of patient postal districts submitted to the Vaximap service between January 2021 and May 2022. Grey denotes no data. Figure produced by the authors using Tableau Public https://public.tableau.com/app/discover.

Figure 3 shows the geographic distribution of uploaded patient locations within the UK, for a subset of the complete dataset. Use of the service has been concentrated in England and Wales; Scotland has seen very little use.

**Fig. 4:**
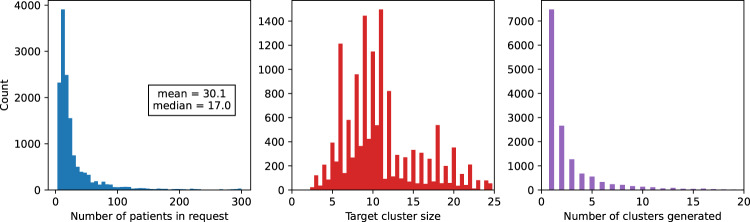
Histograms of user request characteristics. Left: total number of patients uploaded in request. Centre: target cluster size for request. Right: number of clusters generated for request.

Figure 4 shows summary statistics of uploaded user requests. Users uploaded a median of 17 and mean of 30 patients per request, with a target cluster size of around 10. Though a handful of users uploaded the maximum limit of 300 patients, a substantial minority uploaded 10 or so patients, resulting in a single cluster being generated.

### Estimated time and cost savings

Using a threshold of less than 21 days between repeated uploads of the same patient postcodes, 4003 repeat requests were detected. Excluding these repeats, the total dataset was reduced from 14,359 requests covering 432,856 patients to 10,356 requests covering 285,453 patients. The latter figure represents the best estimate of the number of actual home visits that have been performed using the service.

Applied to the full dataset including repeats, the survey-derived lookup times and routing times of 36.4 s and 4.8 s per location, respectively, yielded an estimate of total time savings in planning of 4834 h, equivalent to 120 weeks at 40 h/week.

Applied to the full dataset without repeats, an empirical model of human performance on the TSP yielded an estimate of the total savings in distance travelled of 44,200 km. Assuming a mean travel speed of 50 km/h or 30 mph, this is equivalent to 880 h, or 22 weeks at 40 h/week.

Combining all savings yields a total of 5700 h of practitioner time, equivalent to 142 work weeks or 3 work years. Using mean salary estimates of £38,000 and £32,000 for a practice manager and community nurse respectively (taken from www.glassdoor.com), these time savings can be converted into a cost saving of approximately £110,000.

## Discussion

Vaximap is a simple and easy-to-use solution for optimising vaccine delivery to housebound patients. It has seen widespread use during the UK’s Covid-19 vaccination campaign, reaching 50% of the target patient population (assuming a patient population of 500,000 and using a figure of 285,000 non-repeated visits planned on the site), and yielded both time (3 years of practitioner time) and cost (approximately £110,000) savings. One user in Plymouth reported doubling their rate of delivery by using the service, demonstrating that Vaximap was able to reduce the burden on the healthcare system by simplifying the execution of a complex task and quickly removing vulnerable patients from the unvaccinated population. In turn, the time savings obtained allowed primary care staff to focus on their other care responsibilities.

Harder to quantify are the savings in a cognitive load of automating the complicated process of route planning, but direct feedback from users (published on the Vaximap website) frequently touched on the “frustration” inherent to such a tedious and labour-intensive task. This is especially relevant given the small number of users that uploaded requests containing the maximum allowed number of 300 patients; manually planning a route for this number would be wholly unreasonable. These large requests also serve to illustrate the unequal demand for, and provision of, primary care across the country^7^: a GP surgery looking after hundreds of housebound patients faces very different challenges to one looking after a handful.

Given that Vaximap is simplistic compared to existing TSP or VRP solutions that are in the public domain (some of which are open source, for example, the VROOM project https://github.com/VROOM-Project), it is reasonable to question why this technically inferior solution saw widespread uptake. In the opinion of the authors, three reasons can be given. Firstly, GPs were involved throughout the development of Vaximap and it was therefore tailored exactly to their requirements. For example, the addition of walking directions (alongside driving) was made in response to requests from GP surgeries in urban areas. The low level of sophistication was the minimum sufficient to meet these requirements. Conversely, the sophistication of existing solutions may have been a barrier to their uptake; the fact that GPs rapidly adopted this solution indicates that they were either unaware of existing solutions or did not know how to access and operate them. This shows the importance of interacting directly with users before and during development to ensure their requirements are met, which is pertinent in light of the expectation that digital technologies will play an ever-greater role in primary care^14^. Secondly, uptake was driven via word-of-mouth within informal GP networks (aided by the fact the service could be presented as “made-to-measure” for them). It is believed that this was particularly important to overcome the data-protection concerns that were repeatedly raised during roll-out: once some GPs were using the service, it was more acceptable for others to follow suit. Thirdly, it was made available online as a public-facing website, free of charge, with a deliberately basic user interface. By way of illustration, patient postcodes may only be uploaded via an Excel spreadsheet, as GP feedback indicated that at a minimum all users would know how to export patients from their patient management systems to Excel. It is also important to note that at no stage was user training for the service provided (nor could it be), which is in stark contrast to the vast majority of existing medical software. This necessitated the constant focus on simplicity and ease-of-use; users had to figure out the service for themselves in a very short period without outside help.

There are multiple aspects of the vaccination problem that Vaximap does not account for. The vaccines available at the time of writing have different refrigeration and preparation requirements, detailed in Table 1. Vaximap makes no attempt to account for these and it is left to the end-user to observe them. This is readily achieved by comparing the estimated travel time for each route with the storage requirements of their particular vaccine. Similarly, the possibility of human error or wastage (i.e., that one or more doses from a vial are not used) is left to the user to manage. This is because accounting for error or wastage would require a statistical modelling approach based on previously observed wastage rates; at the outset of the vaccination campaign such data was not available. Nevertheless, a user could easily (if crudely) account for this by setting a cluster size smaller than the number of doses in a vial, assuming other recipients could be found for the excess doses. It should also be noted that numerous grass-roots efforts sprang up to minimise vaccine wastage by re-allocating spare doses to the public at the end of each day (for example, *Vite Ma Dose* in France); it is reasonable to assume GPs re-allocated spare doses left over from housebound vaccinations in this manner.

**Table 1:** Storage and preparation requirements for the four most common Covid-19 vaccines. Data taken from ref. ^26^.

Vaccine	Storage within GP surgery	Preparation	Storage after preparation
Pfizer	1 month between 2 and 8 ^∘^C	Dilute 0.45 ml with 1.8 ml of 0.9% sodium chloride	Up to 6 h below 30 ^∘^C
Oxford-AstraZeneca	6 months between 2 and 8 ^∘^C	No dilution required	Up to 6 h below 30 ^∘^C
Moderna	1 month between 2 and 8 ^∘^C	No dilution required	Up to 6 h below 25 ^∘^C
Novavax	6 months between 2 and 8 ^∘^C	No dilution required	Up to 6 h below 25 ^∘^C

Vaximap is technically simple and could be improved by adopting the strategies used in the operations research literature. As a point of departure, it is known that iterative *k*-means clustering does not always give optimal clusters so it would be desirable to replace this with a more robust approach. Further gains could be obtained by incorporating the more subtle aspects of vaccination, such as cold chain requirements and human error or wastage, but this would require users to provide more information (besides patient postcodes) to the service, which they may not be comfortable doing. Solutions for the VRP with time windows would be particularly suitable for the cold chain requirements. It is easy to foresee a situation in which not all constraints could be respected: for example, in extremely rural areas, the travel time to visit *N* patients in a cluster could exceed the 6 h that the vaccines may be stored at ambient temperature. Particularly in respect of cold chain requirements, care would need to be given to ensure that a more comprehensive solution does not become “software as a medical device”, a possibility that was repeatedly questioned by external parties during development and which would raise regulatory hurdles.

At the time of writing, the service is supporting ongoing booster campaigns, as evidenced by the successive waves shown in Fig. 2. Considering the decay of protection afforded by vaccines as time passes, and the emergence of new variants, Vaximap is well placed to assist with future Covid vaccination efforts. More importantly, the underlying problem that the service addresses will continue to exist long after Covid-19; namely, how to efficiently visit a set of patients subject to some constraint on group size. The technology could therefore be applied in other domains. An obvious example, and one already suggested by multiple users, would be supporting annual flu vaccination campaigns; a more novel example would be supporting district nurses, community nurses and physiotherapists in their daily tasks. Given community healthcare in the UK records around 100 million patient contacts annually with a budget of around £10 billion and one-fifth of the NHS workforce^15^, the cumulative impact of efficiencies obtained at the grassroots level could be substantial.

## Methods

This work has been reviewed by the University of Oxford Medical Sciences Interdivisional Research Ethics Committee and classified as service development and evaluation, which means it does not require ethical review (CUREC application: R79436/RE001).

The term housebound is used here to refer to any individual whose postcode was uploaded to the Vaximap site for the purpose of planning a vaccination. Due to the anonymous nature of the tool, it is not possible to know on what medical basis they are deemed housebound.

### Implementation

The problem solved by Vaximap is posed as finding the shortest routes to visit a set of *N* patients, whilst ensuring any individual practitioner visits no more than *D* patients on a route. It is assumed, but not required, that *D* is set as the number of vaccine doses in a vial (for example, nine for Oxford-AstraZeneca); any number between 3 and 25 inclusive can be used. It follows that the *N* patients must be sorted into *G* = ceiling(*N*/*D*) clusters (rounded up in the case that *D* does not divide perfectly into *N*, in which case exactly one cluster will have size less than *D*). This is an important extra constraint added at the request of a GP, which ensures at most one vial of vaccine will be incompletely consumed. It may be desirable to set *D* smaller than the number of doses in a vial to ensure there is a spare vaccine dose per cluster in case of unforeseen problems out on the road (the most obvious being accidental wastage of a dose due to human error), in which case it would be advantageous to ensure there are willing recipients for any unused excess doses back at the GP surgery.

**Fig. 5:**
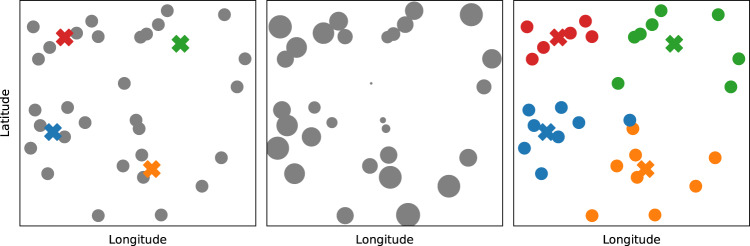
Example clustering of *N* = 30 patients into clusters of *D* = 8. Left: initialisation of four cluster centroids (denoted with crosses). Centre: order of patient assignment, where larger circles indicate priority assignment and will be dealt with first. Patients in the centre of the distribution could be assigned to any cluster with low cost, so are dealt with last. Right: the optimal clustering of patients after assignment.

Patient postcodes uploaded by the user are transformed into latitude and longitude coordinates via the use of Microsoft Bing’s geocoding service. Patients are then clustered so that they are proximal in space according to Euclidean distance, a simple heuristic to minimise the travel time within each cluster. Iterative *k*-means clustering is used to group the patients subject to the constraint on cluster size *D*, which standard *k*-means cannot do^16,17^. Qualitatively, this approach prioritises those patients that are far away from all others to ensure they are assigned to their optimal cluster first, whereas those that are close to the centre of the distribution can be assigned last to any cluster with limited impact on the optimality of the routes. The simplifying assumption implicit to clustering is that all patients are connected via direct paths, which may not be the case in reality (although the subsequent route generation step does not make this assumption). The clustering is however robust to patients that are co-located; i.e., if two or more patients share the same address, they will be counted as distinct locations, ensuring that the constraint on cluster size is not violated. The clustering process is illustrated in Fig. 5.

After cluster assignment, the final step is to determine the optimal order in which to visit the patients of each cluster. This is achieved via Microsoft Bing’s mapping API, making use of the optimise waypoints facility. This uses a proprietary algorithm to perform the optimisation within clusters, accounting for road speeds and traffic levels at the time of route generation (albeit not at the time when the route will be followed, which is not known ahead of time). The Bing maps API imposes a limit of 25 patients per cluster. Optimise waypoints requires a start and end point for each cluster’s route to be specified in advance, after which it will optimise the order of points in between the two. In the case where the user has specified a fixed start and end point for all routes (i.e., their GP surgery), a conventional TSP is recovered. In the case where no fixed start and end point is given, a VRP is recovered and it is necessary to algorithmically specify start and end locations for each cluster’s route (the API does not select them). The heuristic used in this work is to specify the two patients furthest apart from each other on the basis that all other patients will approximately lie on the line connecting them. In either case, the Bing maps API handles the optimisation for the order of intermediate locations. After route generation, the user is returned the patient clusters, the optimal driving or walking directions within each cluster, and travel time estimates.

### Dataset analysis

Since the launch of the Vaximap service in January 2021, a database of service usage has been accumulated (in accordance with the website’s privacy policy). From each user request (corresponding to a single upload of patient postcodes and the routes that result), the following data are retained: time and date; the number of patients; requested cluster size; relative distances between patients (not absolute locations); and transport mode. For approximately half of requests, counts of patient postal districts were also retained (this is the first part of a postcode, for example OX1). Typical postal districts have in excess of 10,000 patients ^18^, so this information is geographically non-precise. As of October 2022, the dataset comprises approximately 14,350 requests for 433,000 patients; postal district information is available for 8000 of the requests.

The objective of this analysis was to estimate the time savings yielded by Vaximap and to identify high-level trends in how the service has been used. The savings arise in two ways: firstly, when planning a route to visit patients; and secondly when following an optimal route instead of a sub-optimal one. The following analyses were performed.

In the course of development, it was noted that users sometimes uploaded the same set of patients multiple times in quick succession, with slightly different parameters. It is suspected that such behaviour reflects a learning process on the part of users who were familiarising themselves with the site. For some of the analyses detailed below, such repeat requests (defined by a request less than 21 days after a previous identical request) were removed. It was not desired to remove repeats separated by more than 21 days, however, as these could represent genuine healthcare use cases, though they may not correspond to repeat Covid-19 vaccinations if separated by only a few weeks.

Summary metrics of the dataset were explored via histograms of the number of patients uploaded per request, the number of clusters the request was split into, and the number of patients per cluster. The time-series nature of the data was explored by plotting the number of user requests per day, and the total number of patients across all requests per day. Finally, the geographic distribution of the data was exploited by plotting the cumulative number of patients across all requests in each UK postal district for the subset of data with this information. These analyses were performed on the dataset including repeats.

The time taken for humans to plan solutions for the TSP is remarkably quick and scales linearly with the number of locations to be visited^19–21^. The problem solved by Vaximap is subtly different to the conventional TSP investigated in the literature because users start with a text-based list of addresses (i.e., from a patient database) as opposed to a visual representation. This difference is important in light of the consensus view that “humans require a visual representation of the problem” in order to solve it effectively^21^. The time taken to plan routes in the absence of Vaximap can therefore be split into two components: a lookup time to generate a spatial representation of the problem, and a routing time to actually plan the routes using this representation.

**Fig. 6:**
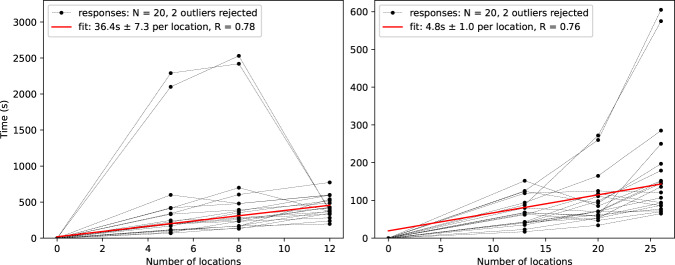
Regressions on survey responses for look up time (left) and route generation time (right). In both cases, two survey responses were deemed outliers and excluded from the regression. The two extremely high response times came from respondents who used a physical map to complete the task (as opposed to a digital service such as Google Maps).

A survey that investigated these two contributions separately was conducted across 20 volunteers. These comprised 8 females, 12 males, mean age 45 years with standard deviation 19; 2 individuals were clinicians; none worked in the field of logistics or had other route-planning experience through 6 had occupations that are explicitly scientific, computational or data-intensive. To estimate lookup time, respondents were asked to identify on a map the location of 5, 8 and 12 sites (identified by their postcodes). To estimate routing time, respondents were asked to propose the optimal route to visit 13, 20 and 26 pre-labelled sites in 2, 3 and 4 routes respectively, where no individual route could exceed 7 sites (which represents the constraint of a fixed cluster size). Linear regression on the completion times yielded the following estimates for the time taken to perform these tasks manually: 36.4 seconds/location for lookup time (*R* = 0.78), and 4.8 s/location for planning time (*R* = 0.76), both of which are illustrated in Fig. 6. Thus, for a 5-site route, we would expect 182 s of lookup time, and 24 s of planning time, totalling 206 s. The objective of this survey was *not* to investigate the optimality of human TSP solutions, for which the existing literature is comprehensive.

The determined coefficients were multiplied across the entire dataset, including repeats, to obtain estimates of the time saved in planning. Repeats were included in this analysis as it was assumed users had reasonable cause for them (for example, experimenting with different cluster sizes and observing the differing travel times that result before making a decision). Finally, a penalty of 30 s per user request was applied to account for the approximate time it would take for a user to upload patient postcodes, choose the appropriate parameters and generate routes.

**Fig. 7:**
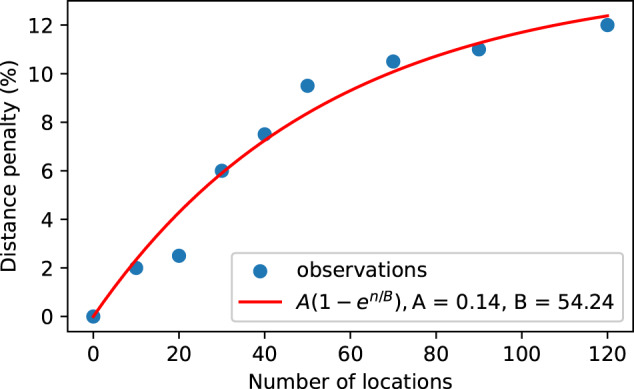
An empirical model of human performance on the TSP, taken from Dry’s review^20^. The individual observations from that work are reproduced here; a curve fit of the form *p*(*n*) = *A*(1 − *e*^*n*/*B*^) was performed to yield a smooth model for the penalty *p*(*n*). The penalty is <10% for problems sized up to around 60 locations.

The optimality of human solutions to the TSP has been investigated extensively^19,21–23^. These are often remarkably close to optimal for small problems and scale well for larger problems (*n* > 50 locations). The empirical model of performance given in Fig. 2b of Dry’s review^20^, reproduced below in Fig. 7, was used in this work to approximate the extra distance penalty *p*(*n*) of human solutions compared to Vaximap’s solution. The approximation is imperfect in respect of the fact that the problem solved by Vaximap is sometimes a VRP; however, given that the majority of Vaximap-generated routes were for *n* ≤ 25 patients, for which the empirical model shows the penalty to be <5%, the ultimate difference in optimality arising due to this approximation should not be substantial. For each user request in the non-repeated dataset, the total (closed) length of the Vaximap-generated routes *L*_*vm*_ was calculated using the following method. The dataset contains the relative latitude and longitude coordinates for each set of uploaded patients. The clustering and optimal order in which to visit the patients are also recorded. Each set has been shifted such that the centroid lies on the global origin (0,0), a non-reversible transformation that obscures the true location of the patients to preserve data privacy. In order to calculate the length of routes between patients, it is necessary to calculate the pair-wise distances between patients. To do so, the patients are first shifted onto the UK by adding (53, −1.2), the approximate mid-point of the UK, to their coordinates. The coordinates are then transformed from latitude and longitude into a Cartesian metric coordinate system (EPSG:3857). From this, all pair-wise distances can be found by calculating the distance matrix for the patients, and finally closed route lengths are found by summing individual pair-wise distances as dictated by the optimal route orders. In the real world, the shortest possible path between two points is rarely straight. The calculation of the distance matrix will thus give an artificially small estimate of total route length by assuming the existence of straight line paths between all patients. In order to correct this discrepancy, a multiplication by a *detour index* (ratio of real world distance to straight line distance) of 1.4 was performed^24^. The approximate length of the human solution *L*_*h*_ to the same problem was then obtained by multiplication with a scaling factor of (1 + *p*(*n*)) drawn from Fig. 7. In order to convert distance savings into time savings, a mean driving speed of 50 km/h or 30 mph was assumed^25^. Repeats were not included in this estimate: though a user may have planned the same route twice in one week, they are unlikely to have actually undertaken it twice.

## Data Availability

The data on which analysis was performed may be accessed at Zenodo via the doi: 10.5281/zenodo.7330382.

## Code availability

The analysis was performed using Python 3.8.13, Pandas 1.4.1, SciPy 1.8.1, Numpy 1.22.3 and PyProj 3.3.1. The analysis code may be accessed at GitHub: https://github.com/tomfrankkirk/vaximap_paper/tree/1295628904cc636e13e86989a7c3d3ad69f746bb
